# Cardiovascular diseases prediction by machine learning incorporation with deep learning

**DOI:** 10.3389/fmed.2023.1150933

**Published:** 2023-04-17

**Authors:** Sivakannan Subramani, Neeraj Varshney, M. Vijay Anand, Manzoore Elahi M. Soudagar, Lamya Ahmed Al-keridis, Tarun Kumar Upadhyay, Nawaf Alshammari, Mohd Saeed, Kumaran Subramanian, Krishnan Anbarasu, Karunakaran Rohini

**Affiliations:** ^1^Department of Advanced Computing, St. Joseph's University, Bengaluru, Karnataka, India; ^2^Department of Computer Engineering and Applications, GLA University, Mathura, Uttar Pradesh, India; ^3^Department of Mechanical Engineering, Kongu Engineering College, Perundurai, Erode, Tamil Nadu, India; ^4^Department of VLSI Microelectronics, Saveetha School of Engineering, SIMATS, Chennai, Tamil Nadu, India; ^5^Faculty of Science, Princess Norah Bint Abdulrahman University, Riyadh, Saudi Arabia; ^6^Department of Biotechnology, Parul Institute of Applied Sciences and Centre of Research for Development, Parul University, Vadodara, India; ^7^Department of Biology, College of Science, University of Hail, Hail, Saudi Arabia; ^8^Centre for Drug Discovery and Development, Sathyabama Institute of Science and Technology, Chennai, Tamil Nadu, India; ^9^Department of Bioinformatics, Saveetha School of Engineering, SIMATS, Chennai, Tamil Nadu, India; ^10^Unit of Biochemistry, Centre of Excellence for Biomaterials Engeneering, Faculty of Medicine, AIMST University, Semeleing, Bedong, Malaysia; ^11^Centre for Excellence for Biomaterials Science AIMST University, Semeling, Bedong, Malaysia; ^12^Department of Computational Biology, Saveetha School of Engineering, SIMATS, Chennai, Tamil Nadu, India

**Keywords:** cardiovascular disease, AI-based technologies, internet of things, machine learning, computational method

## Abstract

It is yet unknown what causes cardiovascular disease (CVD), but we do know that it is associated with a high risk of death, as well as severe morbidity and disability. There is an urgent need for AI-based technologies that are able to promptly and reliably predict the future outcomes of individuals who have cardiovascular disease. The Internet of Things (IoT) is serving as a driving force behind the development of CVD prediction. In order to analyse and make predictions based on the data that IoT devices receive, machine learning (ML) is used. Traditional machine learning algorithms are unable to take differences in the data into account and have a low level of accuracy in their model predictions. This research presents a collection of machine learning models that can be used to address this problem. These models take into account the data observation mechanisms and training procedures of a number of different algorithms. In order to verify the efficacy of our strategy, we combined the Heart Dataset with other classification models. The proposed method provides nearly 96 percent of accuracy result than other existing methods and the complete analysis over several metrics has been analysed and provided. Research in the field of deep learning will benefit from additional data from a large number of medical institutions, which may be used for the development of artificial neural network structures.

## 1. Introduction

Cardiovascular disease (CVD), which is the leading cause of death globally, has become a significant problem in public health all over the world. As a result, patients, their families, and the governments of these countries have incurred substantial socioeconomic expenses. Patients at high risk for CVD can be identified by prediction models that use risk stratification. After that, measures that are tailored to this group, such as dietary changes and the use of statins, can help reduce that risk and contribute to the primary prevention of CVD (1).

Several guidelines for the evaluation and management of CVD have suggested using predictive models as a means of identifying patients at high risk and assisting with clinical decision-making. The Pooled Cohort Equations and the Framingham CV risk equation6, for example, have both been subjected to independent evaluations in a variety of populations; however, the findings indicated that both of these equations were only weakly discriminating and had a poor level of calibration (2).

As a direct consequence of this, the predictive power of the majority of the models that are now in use is restricted, and there is room for advancement. For instance, the assumption of linearity is necessary for logistic regression, while the assumption of predictor independence is necessary for the Cox proportional hazard model (3).

In the area of study pertaining to the cardiovascular system, machine learning (ML) algorithms have been demonstrated to be extremely helpful predictors. They are more adept than standard statistical models at capturing the complex interactions and nonlinear linkages that exist between the variables and the results (4). Several different investigations (5–15) came to the conclusion that random forests (RF) and support vector machines (SVM) performed better than traditional models.

Cardiovascular diseases such as coronary artery disease (CAD), atrial fibrillation (AF), and other cardiac or vascular ailments continue to be the leading cause of death in the world (10). As people living standards improve and their stress levels continue to rise, the number of people who suffer from CVD is growing at an alarming rate.

**Figure 1:**
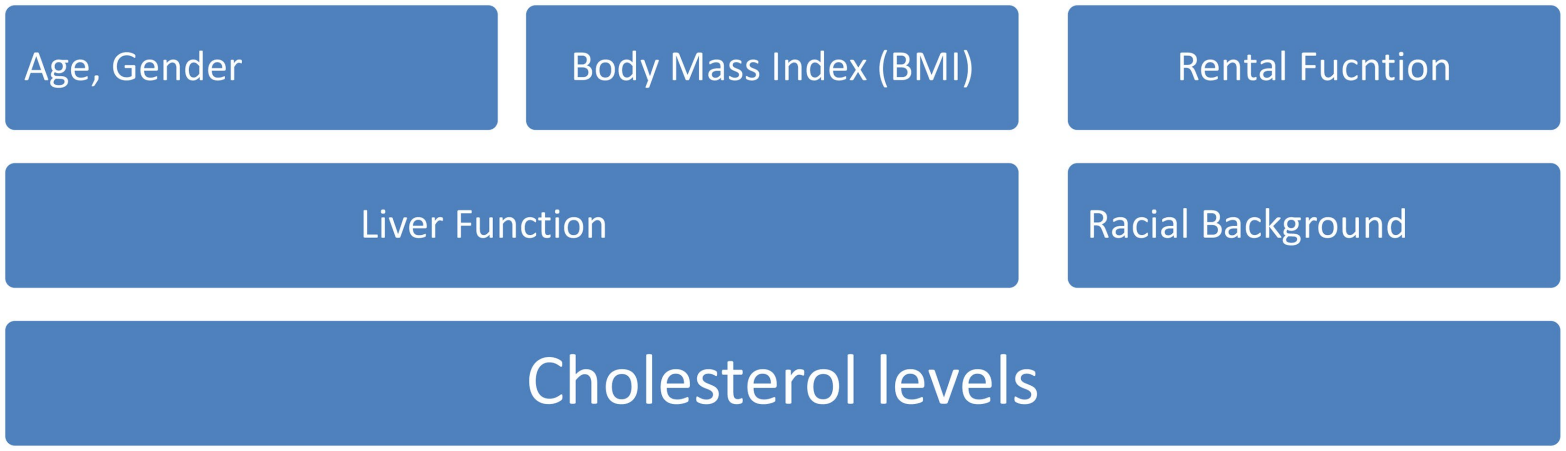
Several factor influencing incidence in cardiovascular disease.

According to the most recent estimations (16, 17), CVD will be responsible for the deaths of about 23 million people by the year 2030. Infarction of the myocardium, atrial fibrillation, and heart failure are all instances of different types of CVD (18, 19). The incidence of cardiovascular disease can be influenced by a number of factors, including racial or ethnic background, age, gender, body mass index (BMI), height, and length of torso, as well as the outcomes of blood tests that evaluate factors such as renal function, liver function, and cholesterol levels (20, 21) which is shown in Figure 1.

The development of a wide variety of health problems can be influenced by the complex interactions that take place between these factors. Standard statistical approaches are incapable of accounting for all of the intricate causal links that exist between risk factors because there are so many of them (22, 23). In this day and age of big data, the Internet of Things (IoT) has been shown to be of critical importance. It has made it possible for patients to use smart drugs and smart bracelets to monitor and collect accurate data during a pandemic (24).

Researchers are employing artificial intelligence (AI) in an effort to mine new medical information that can be used by clinicians to better understand the symptoms of various diseases and, as a result, make more informed decisions for patients (25). This comes as the prevalence of data from the internet of things (IoT) grows within healthcare systems. In order to investigate previously unknown risk factors, current efforts to standardise medical data, and efforts to organise national health screening data (26–28), we will first standardise medical data. These risk variables may have a correlation with the occurrence of the disease, which means that they could offer insights into the mechanisms underlying the disease. Furthermore, accurate disease incidence prediction models necessitate the analysis of large amounts of data (29, 30). The use of artificial intelligence (AI) and massive amounts of data in the prediction of CVD models is becoming increasingly common.

The main contribution and novelty of this research is mentioned below:

- To extract a total of 11 distinct characteristics from the dataset.
- After that, we started by normalising the data and then proceeded to divide the Heart dataset into training and testing sets using an 8:2 split.
- Afterwards, the incorporated GBDT is utilised in the SHAP method for the purpose of selecting features.
- It helps to construct a stacking model consisting of a base learner layer in addition to a meta learner layer.
- Finally, we will achieve the results over several performance metrics analyses and method in the stacking model.

## 2. Background

Weng et al. (31) tested four different models using clinical data from over 300,000 homes in the United Kingdom. According to the findings, NN was the method that produced the most accurate CVD prediction results for the larger amount of data that were analysed.

The three traditional machine learning models that were tested and evaluated by Dimopoulos et al. (32) based on ATTICA data with 2020 samples for the little CVD dataset were K-Nearest Neighbour (KNN), Random Forest (RF), and Decision Tree. When compared, RF was shown to have produced the best results by using the HellenicSCORE tool, which is a calibration of the ESC Score.

In view of the growing popularity of machine learning techniques in IoT applications, Mohan et al. (15) have proposed a hybrid HRFLM strategy as a means of further improving the accuracy of the model predictions in light of the aforementioned popularity of machine learning methods.

An IoT-ML method was investigated by Akash et al. (33) with the goal of predicting the condition of the cardiovascular system in the human body. The algorithm model uses machine learning (ML) techniques to compute and predict the patient cardiovascular health after it has obtained essential data from the human body. This data include the patient heart rate, ECG signal, and cholesterol.

Within the framework of Yang et al. (34) examination of local locations with separate prediction models, LR was utilised to evaluate 30 cardiovascular disease-related characteristics utilising more than 200,000 high-risk participants in eastern China. The results of the experiments led to the development of an RF model that is more suited to eastern China.

For the first time in the study of CVDs, the idea of a stacking model was presented for the very first time by Yang et al. (35). The data on air pollution and weather were considered in order to have a better understanding of how the stacking model influences the daily hospitalisation rate for CVDs. In order to assist in the construction of the stacking model, a grassroots level of five basic learners was first constructed.

During this period, digital, fully automated ecosystems as well as cyber-physical systems are fast growing and finding applications all over the world. The creation of smart healthcare, which offers tools and processes for early diagnosis of life-threatening disorders, is one example of the innovative concepts and technical compositions that are being implemented in nearly every business. As the fourth industrial revolution moves towards a society that is more technologically advanced, there is an urgent requirement for additional research into CVD Zheng et al. (36).

## 3. Proposed method

The first thing that needs to be done is to combine the data from the Heart Dataset, which already contains information from Cleveland, Hungarian, and Swizerlang, as well as data from Long Beach VA and Stalog (Heart). From the five sources, we extracted a total of 11 distinct characteristics. After that, we started by normalising the data and then proceeded to divide the Heart dataset into training and testing sets using an 8:2 split. Afterwards, the incorporated GBDT is utilised in the SHAP method for the purpose of selecting features.

In the following stage, we will construct a stacking model consisting of a base learner layer in addition to a meta learner layer. The study uses RF, LR, MLP, ET, and CatBoost classifiers to serve as our base learners. LR is utilised in the role of the meta learner. Finally, the suggested stacking model is assessed with regard to its accuracy, precision, recall, F1 score, and area under the curve (AUC). In order to evaluate the model adaptability to new contexts, we made use of a publicly available dataset known as the Heart Attack Dataset.

The Cleveland, Hungarian, Swizerlang, Long Beach VA, and Stalog (Heart) datasets, together with others from the machine learning repository at the University of California, Irvine (UCI), were combined to form the Heart Dataset. We began with a total of 1,190 samples, and after deleting 272 duplicates, we were left with 918 unique sample datasets. We started with 1,190 samples. The whole Heart dataset is displayed in Table 1, and it consists of 11 features that were taken from five different datasets that contained significant relevant features.

**Table 1:** Heart dataset features.

Feature	Detailed Information
Age	Age of the patient
Sex	Sex of the patient (Male: 0 or female: 1)
Chest pain type	Four chest pain types
	ATA: atypical angina
	TA: typical angina
	ASY: asymptomatic
	NAP: non-angina
Resting BP	Value of blood pressure during fasting (Unit mm hg)
Cholesterol	Concentration of serum cholesterol (Unit mm/dL)
Fasting BS	Value of blood glucose during fasting (1: blood glucose >120 mg/dL, 0: other)
Resting ECG	Resting electrocardiogram
Max HR	maximum heart rate
Exercise angina	Presence of exercise angina
Old peak	ST value decision
ST_Slope	Slope of ST section at the movement peak (up, flat, and down)

### 3.1. Feature select and analysis

It is feasible to increase model performance and save a considerable amount of runtime by selecting the ideal subset of features that have a significant impact on the prediction outcomes. This process is referred to as feature selection, and it is possible to accomplish both of these goals.

The three most common methods for picking characteristics are called filters, wrappers, and embedding. The research we conducted utilised the embedded approach known as GBDT as a means of selecting feature variables. This was due to the fact that embedded techniques offer superior prediction performance compared to filter methods and are noticeably quicker than wrapper methods.

GBDT makes use of an additive model and a forward stepwise algorithm in order to achieve learning. These two components work together to accomplish this. For non-leaf nodes, the significance of the features increases proportionately with the magnitude of the reduction in weighted impurity that occurs during splitting.

Because of this, it is not possible to provide a detailed explanation of the role that each attribute plays in determining the overall accuracy of the predictions made by the integrated GBDT. In order to find a solution to this issue, we make use of a technique known as feature imputation, in which the explanatory model is a linear function of the values produced by feature imputation.


(1) $$l\left(z^{\prime }=\emptyset 0+\sum \mathrm{Ni}=1\emptyset {\mathrm{iZ}}^{\prime }i\right)$$

where N—features; ∅_i_—feature attribute value, and Z′i—feature is valid or not.

The Φ*_i_* value of Equation (1) can be determined by employing a tree-valued estimate methodology (also known as the SHAP method), which is founded on the concepts of game theory and used as the feature attribute values. Below is a formulation for a model *f* and a set *S* of non-zero *Z*′ indices, as well as the conventional spherically valued attribute ∅*_i_* for each feature.


(2) ∅i=∑S∈M{i}|S|!(N−|S|−1)!N![f(S∪{i}−f(S))]

where *M*—input feature set.

It is essential to keep in mind that the SHAP strategy is adapted to the specific context and tailored to individual needs. In contrast to the tree model gain, this method can produce consistent results for global feature attributes. This is an advantage over the tree model gain. In the course of our study, we make use of the SHAP methodology in order to isolate and assess several individual characteristics.

In addition to this, we investigate the ways in which various characteristics interact with one another in order to improve our ability to predict outcomes. We differentiate between the contributions of individual features and the contributions of feature interactions by referring to the former as individual feature contribution and the latter as joint feature contribution Φ*_i,j_*. In the same way as the value, the Shapeley interaction index is calculated using a formula, and the joint feature contribution *i* and *j* can be found by doing the calculation as follows.


(3) ∅i,j=∑S∈M{i}|S|!(N−|S|−Z)!Z(N−1)!∇i,j(S)

When *i* ≠ *j*:


(4) ∇i,j(S)=f(S∪{i,j})−f(S∪{i})−f(S∪{j})+f(S)

where Z represents the indices. *i*,*j* represent the feature contributions. S represents the Shapeley interaction Index.

Equations (3) and (4) in the GBDT model quantify the twinning relationships between joint features. So, when judging the model, you can get a good idea of how the different factors that interact with each other contribute together.

### 3.2. Model building

To the extent that the model predictions are accurate, each individual in the base population has a stronger capacity for learning, and the degree of correlation between them decreases. When the individual learners are already more accurate, a fusion of models will be more successful if the individual learners come from a diverse range of backgrounds. This is the foundation upon which the concept of error-ambiguity decomposition is built.

This suggests that when picking the foundation learners for our organisation, we should take into account the performance of individual learners while also taking into account the distinctiveness of each individual learner. Theoretically, it is conceivable to build layers of the stacking model indefinitely as long as their fundamental classifier is operational. This, of course, results in an increase in the level of complexity represented by the model.

To ensure accuracy while reducing the level of complexity exhibited by the model, we solely employ the stacking model, which is comprised of a two-tiered structure consisting of base learners and meta-learners. As a direct consequence of this, SVM, KNN, LR, and ET were decided upon as the possible models for base learners to utilise in the prediction of CVDs. XGBoost, LightGBM, CatBoost, and MLP were some of the other options that were thought about. Following the selection of the most reliable models as the foundation for our education, we restricted the pool of potential candidates to five people who exemplified a comprehensive representation of the population as a whole. The optuna framework was used in order to get the optimal values for the model parameters.

After running a 5-fold CV, this model may generate a large number of features. 5-fold CV is a technique that is frequently utilised in the first layer of a stacking framework to collect input features for the second layer. This paper employs linear regression (LR) as the classifier for the fusion model predictions since generalised linear regression, also known as GLR, has historically been employed in the second layer of the stacking architecture. Because adjusting the complexity of the output layer of a neural network does not require the employment of more complex functions, this example makes use of functions that are simpler in nature.

The primary learners are the LR, RF, DT, MLP, and CatBoost protocols. At the beginning, we give the training sets eight times as many points as the testing sets. Within the training package that we provide for each of the five foundational learners, we utilise a 5-fold CV. A single base learner is capable of producing five predictions, and each of these five predictions is arranged in a vertical column within a one-dimensional matrix. It possible that the second stage of training will be based on a five-dimensional matrix that been developed using the data of five different learners as its foundation.

When applied to the testing set, the 5-fold CV model is utilised once more to make predictions about our initial testing set, which results in the production of five predictions once more. The base learners can be concatenated into a matrix for the stage second iteration. We use LR on the meta-learner so that it does not become too good at its job. In the second step of the process, we use these predictions to build the final results.

## 4. Results and discussion

The outcomes of the experiments will be discussed here in order to illustrate the benefits of the stacking paradigm that was recommended by us. Python version 3.9.7 was used throughout each and every test. In this investigation, the sklearn 1.0.2 toolbox is used for model prediction. The SHAP 40.0 toolbox is used for feature selection, and the Optuna 2.10.0 framework is used to determine the optimum values for the model parameters which is shown in Table 2. We executed 10 splits of the data set using various random seeds in order to account for the small sample size of this study and the aforementioned randomisation. After doing so, we averaged the results of all 10 experiments.

**Table 2:** Software specifications.

Language	Python Version 3.9.7
Operating system	Windows 11
Tool box for model predicition	Sklearn 1.0.2
Feature selection	SHAP 40.0
Optimum values	Optuna 2.10.0 framework

**Figure 2:**
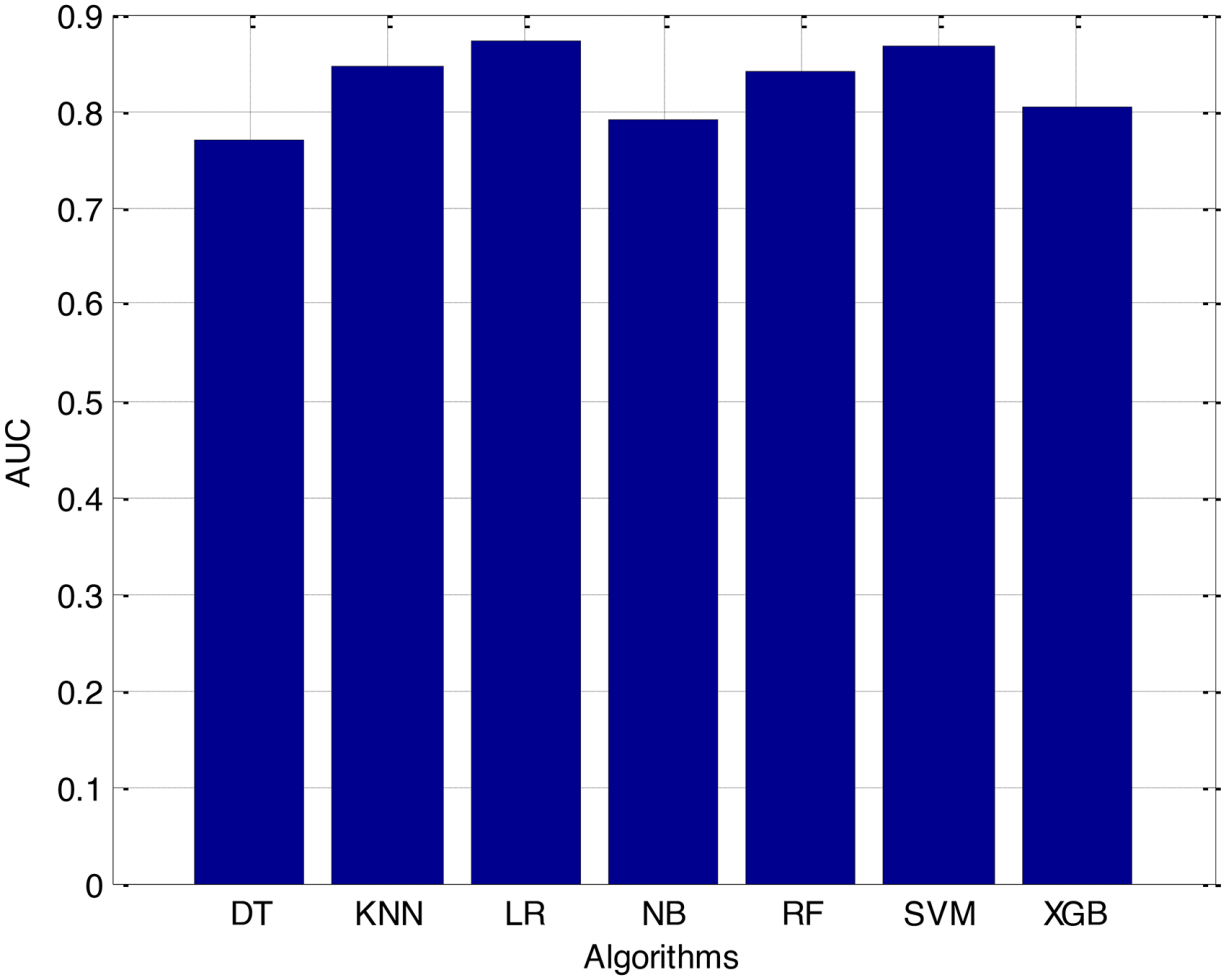
Area under the curve (AUC).

**Figure 3:**
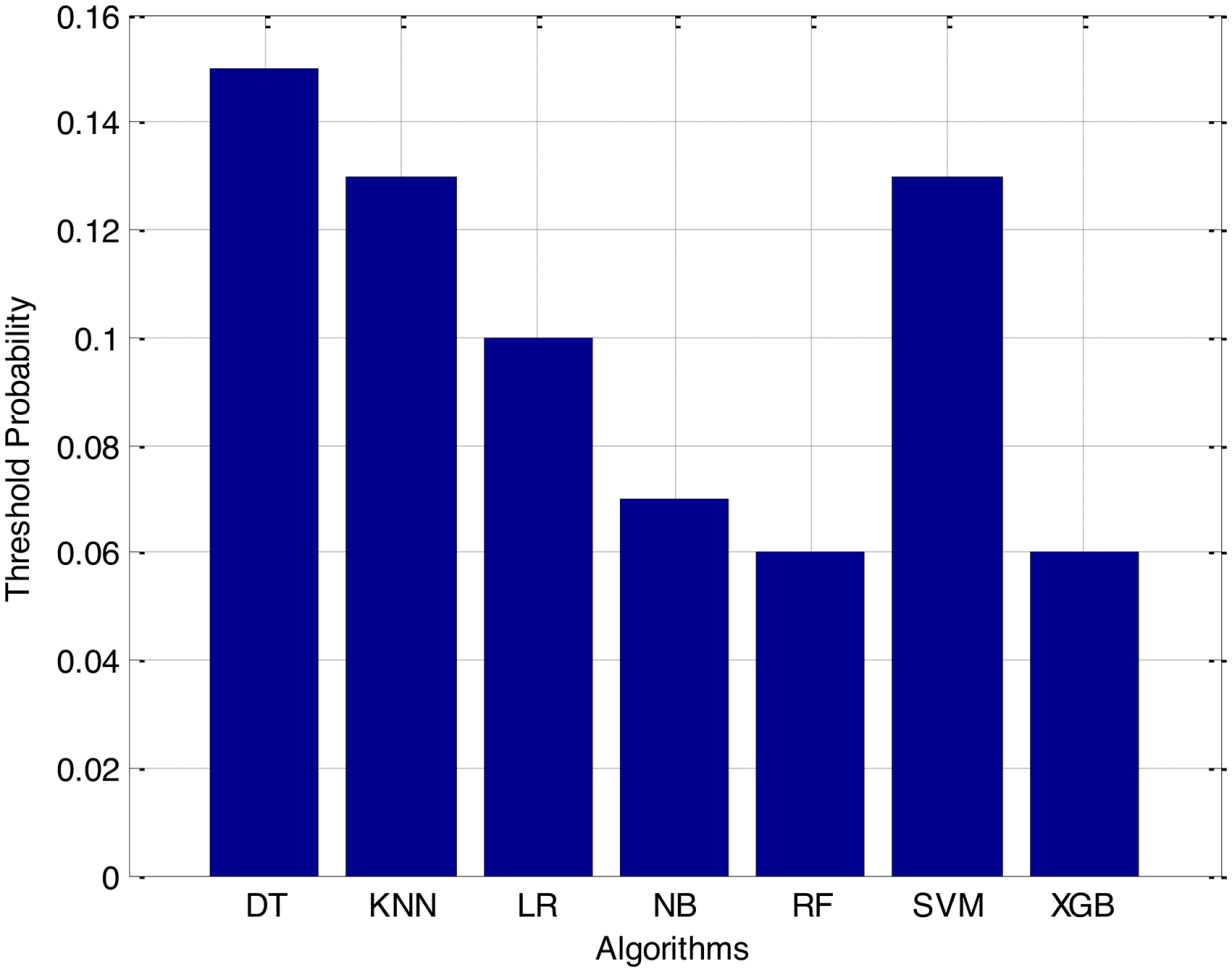
Threshold probability.

**Figure 4:**
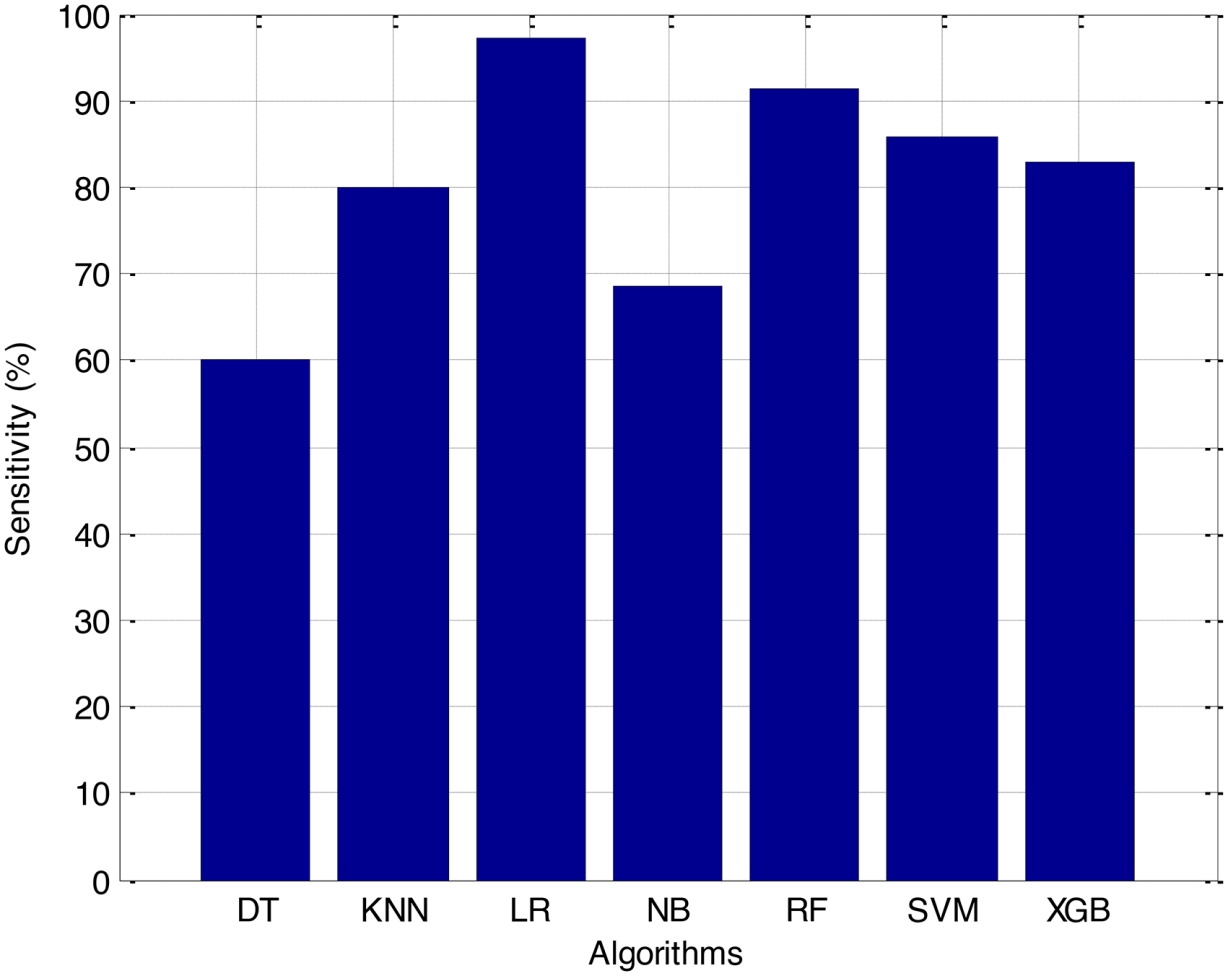
Sensitivity (%).

**Figure 5:**
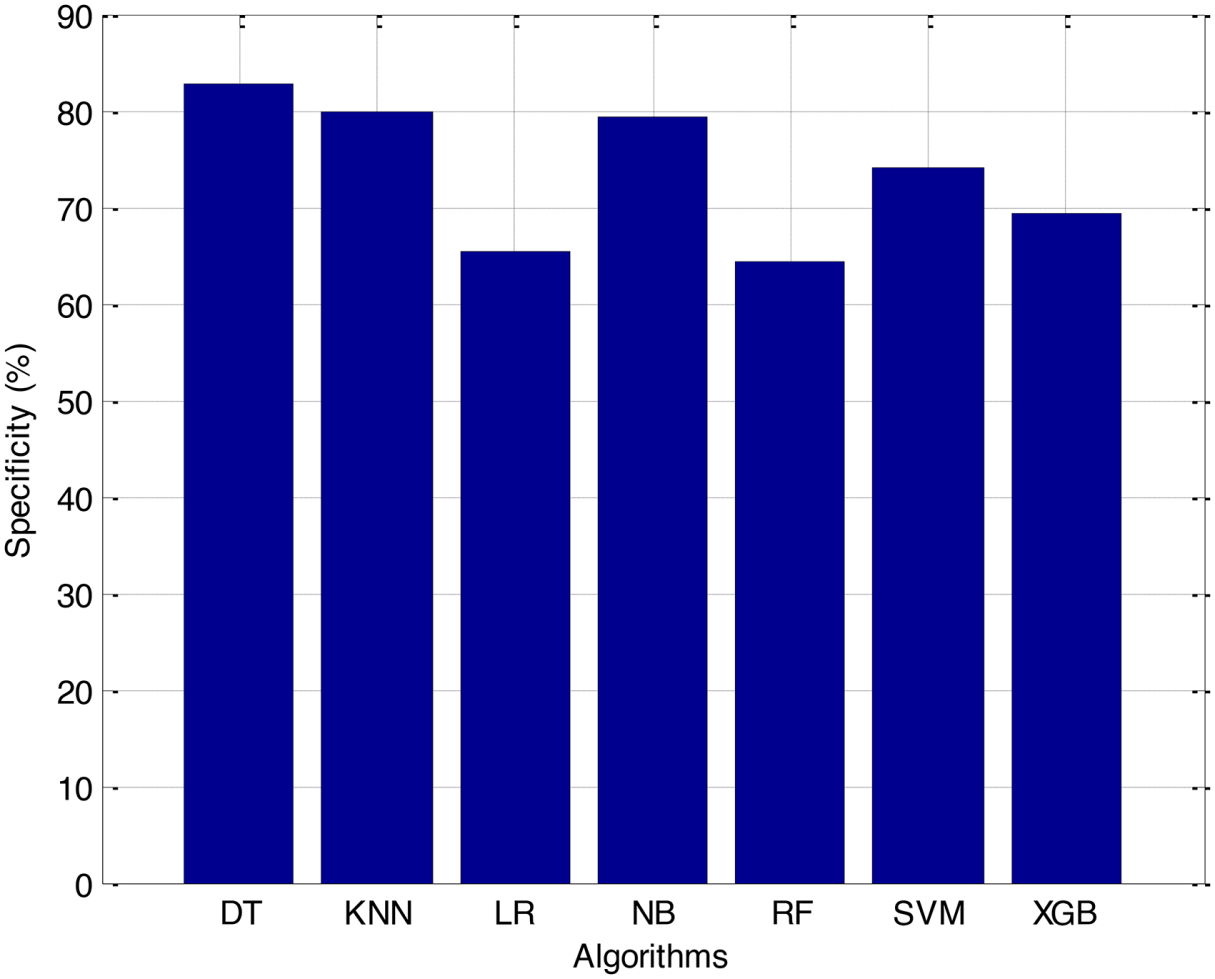
Specificity (%).

Before we started the feature selection process, our dataset contained a total of 11 features. Using the Tree SHAP approach, you are able to determine the contribution value that corresponds to each feature that is contained inside the sample dataset. The ranking of the feature contributions is determined by using the average SHAP value for all of the samples. In accordance with the GBDT model, the contributions of the global features are depicted. The ST Slope and Chest Pain Type have a significant influence on the patient condition (CVD) in patients with cardiovascular disease. In order to cut the model operating time even more, some features that aren’t necessary will have to be eliminated. We chose to adopt a cutoff of 0.02, which led to the elimination of the Resting ECG characteristic while permitting the retention of the other 10 features. We used the AUC to evaluate the performance both before and after the feature selection process. Even though the AUC values of GBDT went down, the drop wasnot substantial at all, and there was not any difference that could be considered statistically significant by performing metrics such as AUC, Threshold, Sensitivity, Specificity which is shown in Figures 2–5.

The incidence of CVD was quite low in this experiment, resulting in poor PPV and NPV values for each of the seven different ML models. Because of this, their therapeutic value may suffer as a result of an increase in the number of false-positive results. The probabilities that were predicted by each machine learning model were unique, and the risk distribution for LR was comparable to that of SVM. Patients who had a CVD episode had estimated risks that were greater, across all ML models, than those patients who had not had a CVD episode. The plots also demonstrated that all ML models overestimated the risks of those individuals who had not experienced any CVD events. This finding suggests that this variable may also affect how well a model predicts what will happen.

It is necessary to have a risk model in order to determine whether individuals have a high probability of developing CVD. We intended to test seven machine learning (ML)-based models to evaluate how correctly they could predict the risk of CVD. The findings demonstrated that each one of them had good to excellent discrimination and that they were all accurately calibrated. When it came to forecasting the risk of CVD, penalised LR performed better than other machine learning models, just like SVM did. The specificity of the SVM was higher than that of the LR, while the LR had a lower level of sensitivity. It is possible that a higher level of specialisation was favoured in this Kazakh Chinese group because the majority of the population was nomadic and there were few medical services available. In addition to this, when taking calibration and DCA into consideration, SVM fared marginally better than LR. Because of this, SVM and LR can be used to find people in this group who are at a higher risk of CVD and to find out if putting risk-mitigation interventions in place for these people will improve their CVD outcomes during the clinical decision-making process.

Linear regression has been widely used in the clinic to construct predictive models due to the ease with which it may be interpreted and its general straightforwardness. In a study aimed at predicting myocardial ischemia, both LR and SVM were shown to have the same level of predictive ability, which was consistent with our findings. A recent and exhaustive study concluded that there is no performance benefit to be gained from using ML in clinical prediction models over using LR. It was determined that when machine learning algorithms were applied to small datasets with a limited number of predictors, LR models might perform better than ML models in terms of overall performance. It is possible that the small sample size and the L1 penalised technique used in this work are to blame for the superior performance of LR in comparison to other machine learning models, with the exception of SVM.

The Support Vector Machine (SVM) is a well-known supervised machine learning approach that has found applications in a wide variety of business sectors. The fundamental idea behind support vector machines (SVM) is to locate the hyperplane that has the capacity to effectively classify the data while also providing the biggest geometric margin. In addition to this, it possesses significant kernel capabilities, which simplify the process of dealing with nonlinear classification issues. The outstanding performance of SVM demonstrates that it is a great tool for tackling classification challenges on small, non-linear, and high-dimensional datasets. This demonstrates that SVM is an excellent tool. In our experiment, we observed that the SVM performed significantly better than other machine learning models.

When it comes to classification, RF is among the most successful ensemble learning strategies that may be used. Its predictions were not nearly as accurate as those generated by the LR and SVM algorithms, which were the other two options. It is likely that the limited number of participants in this study will prevent RF from achieving its full potential as a prediction tool. The concept of variable importance was utilised in order to locate potential indicators of CVD. Some studies suggest that RF may be capable of revealing crucial but undisclosed predictions.

**Figure 6:**
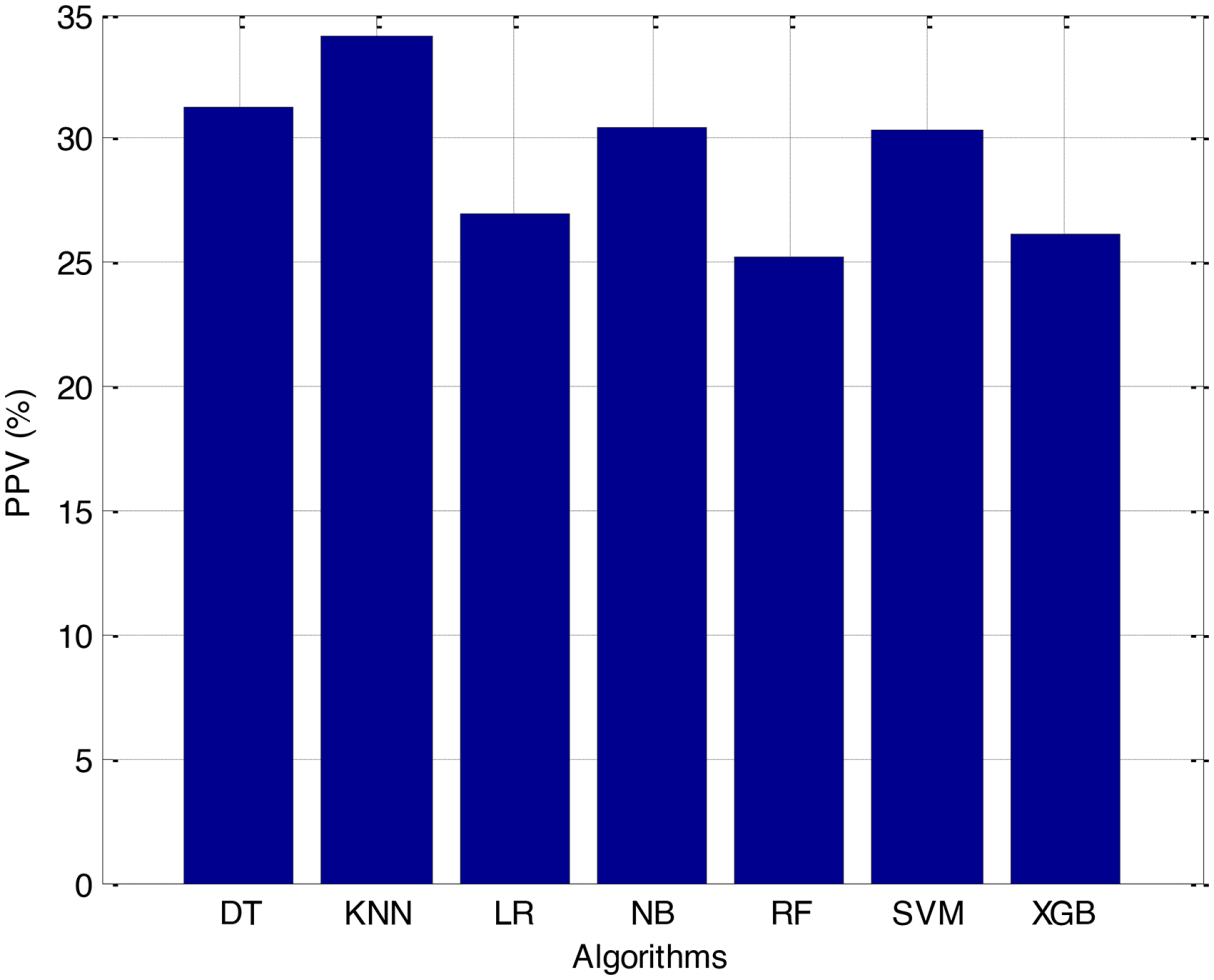
PPV (%).

**Figure 7:**
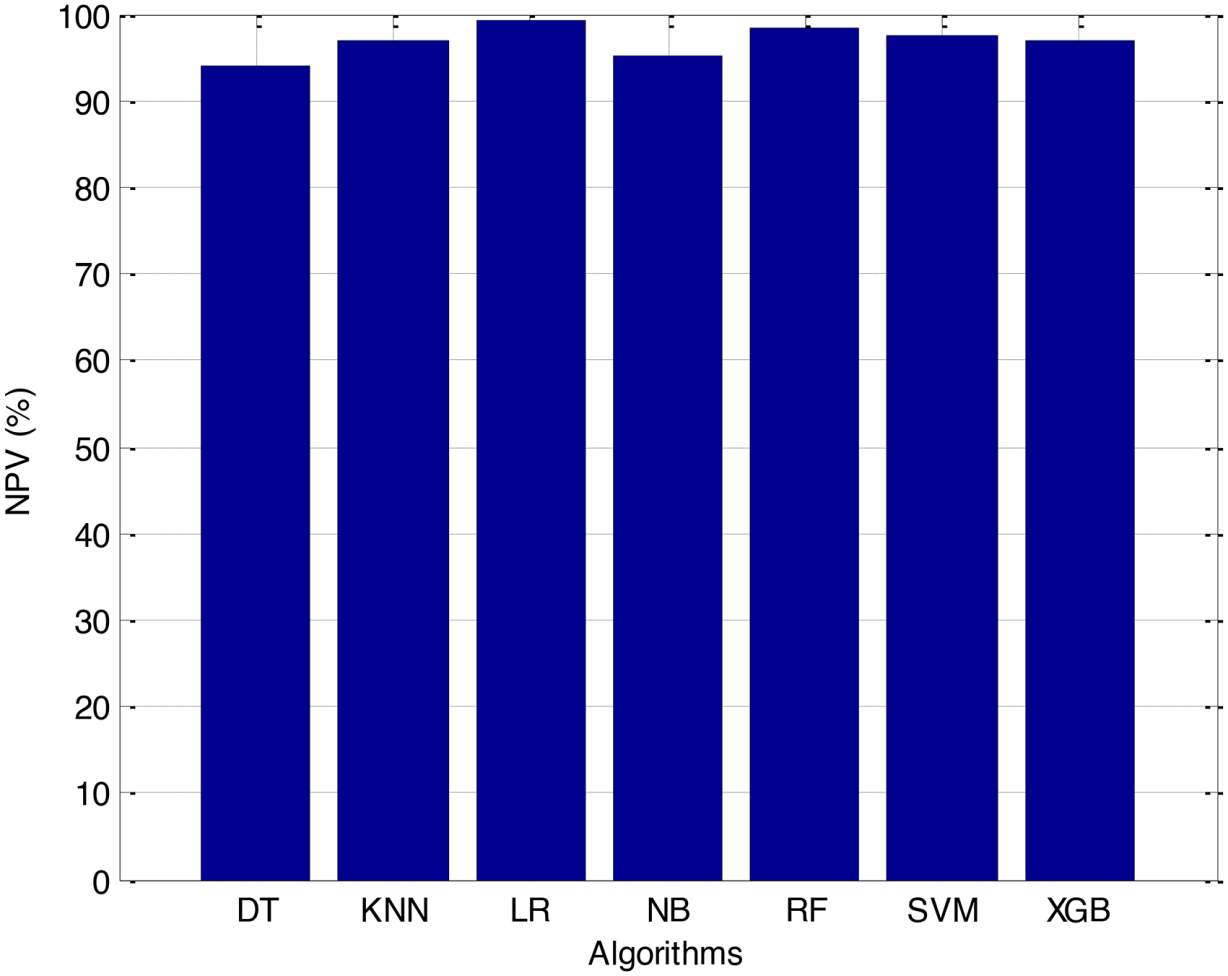
NPV (%).

**Figure 8:**
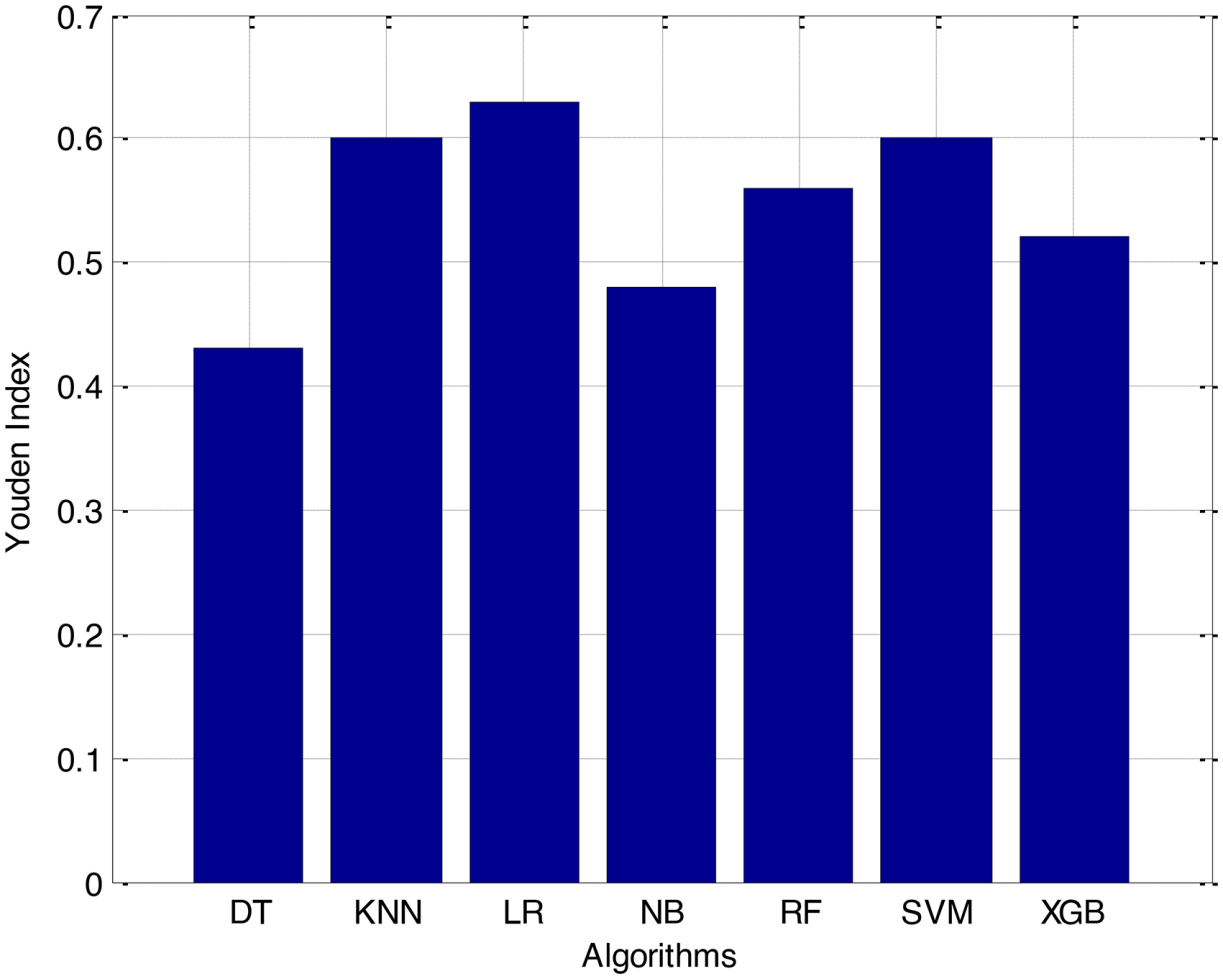
Youden index.

**Figure 9:**
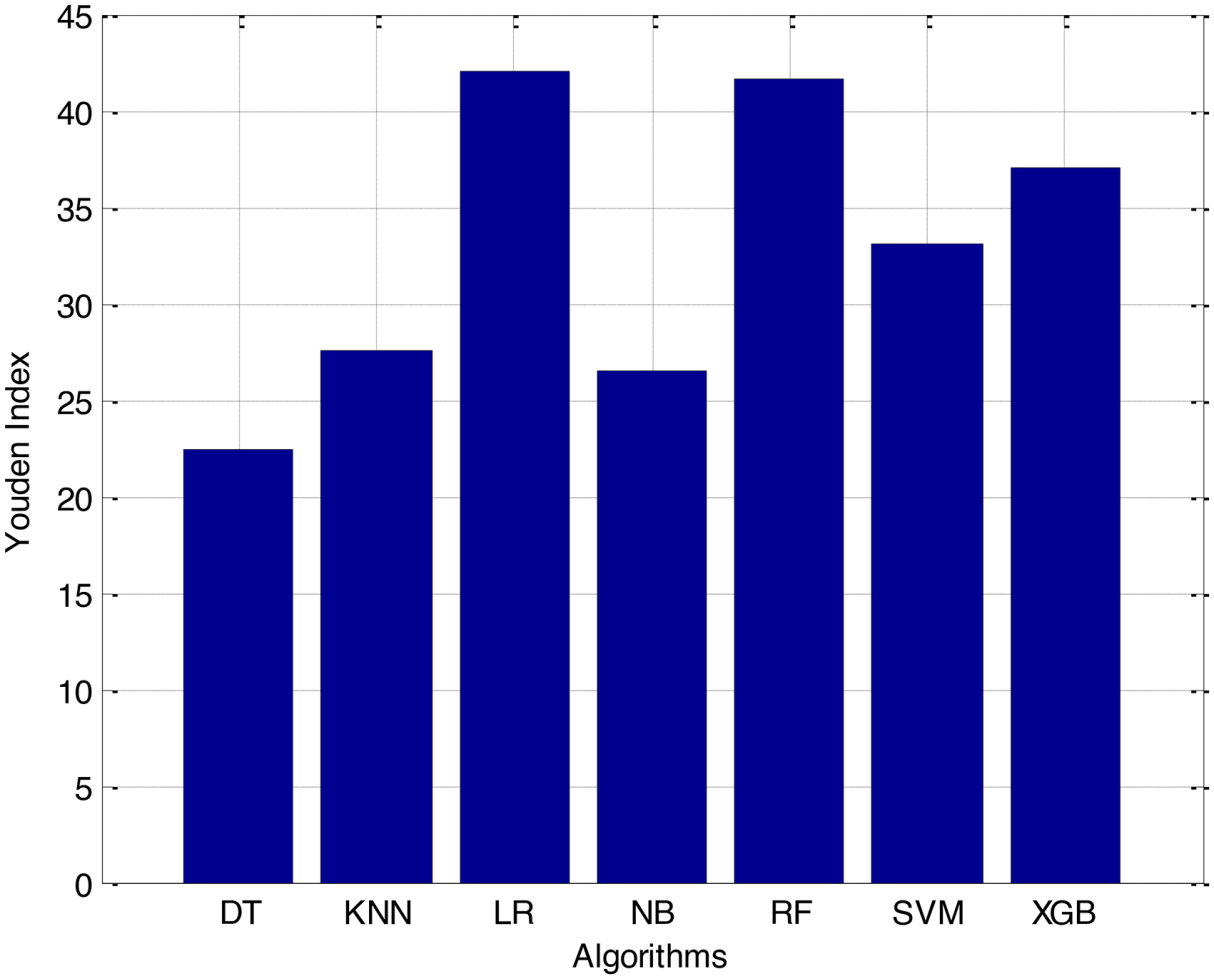
High-risk patients (%).

**Figure 10:**
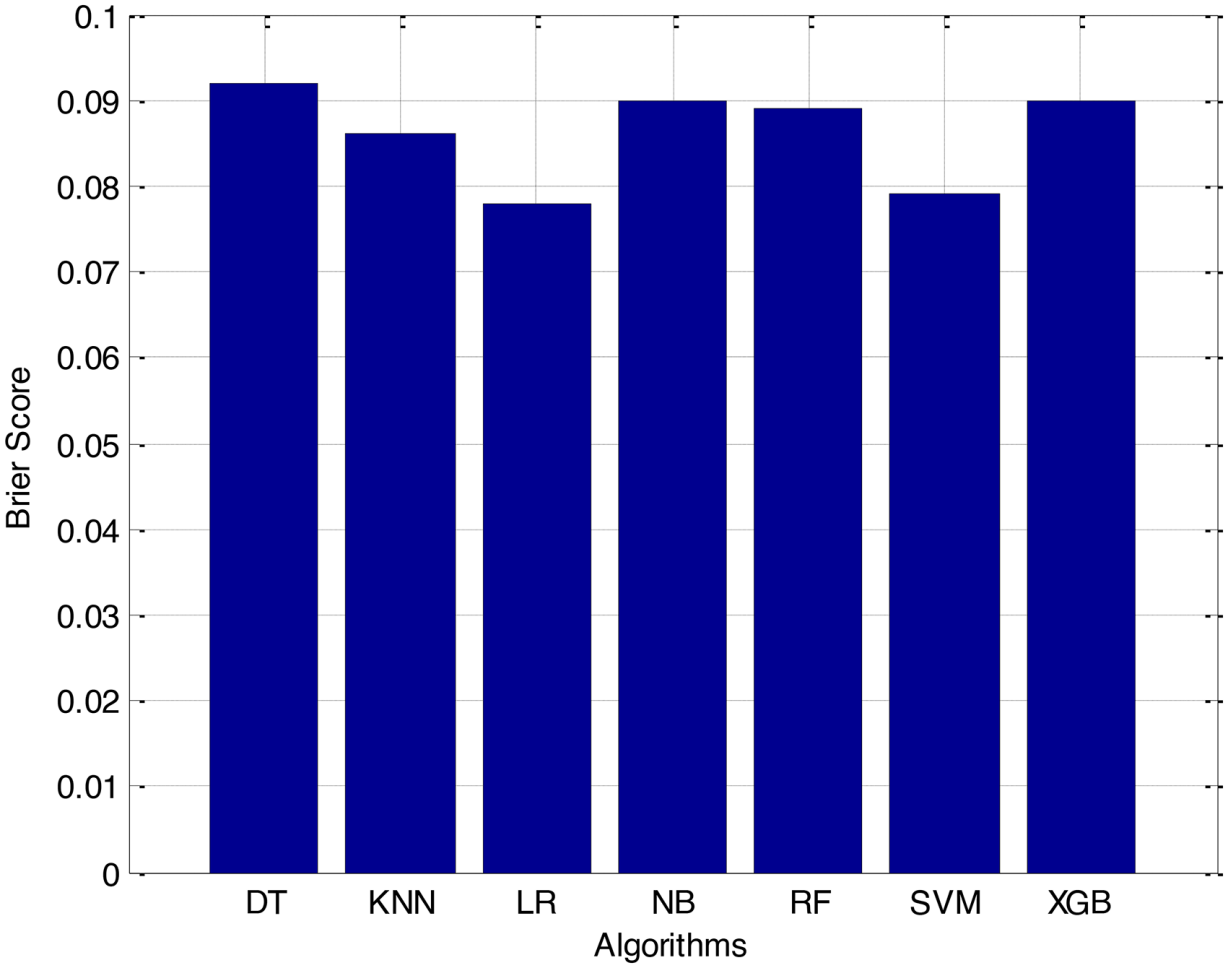
Brier score.

**Figure 11:**
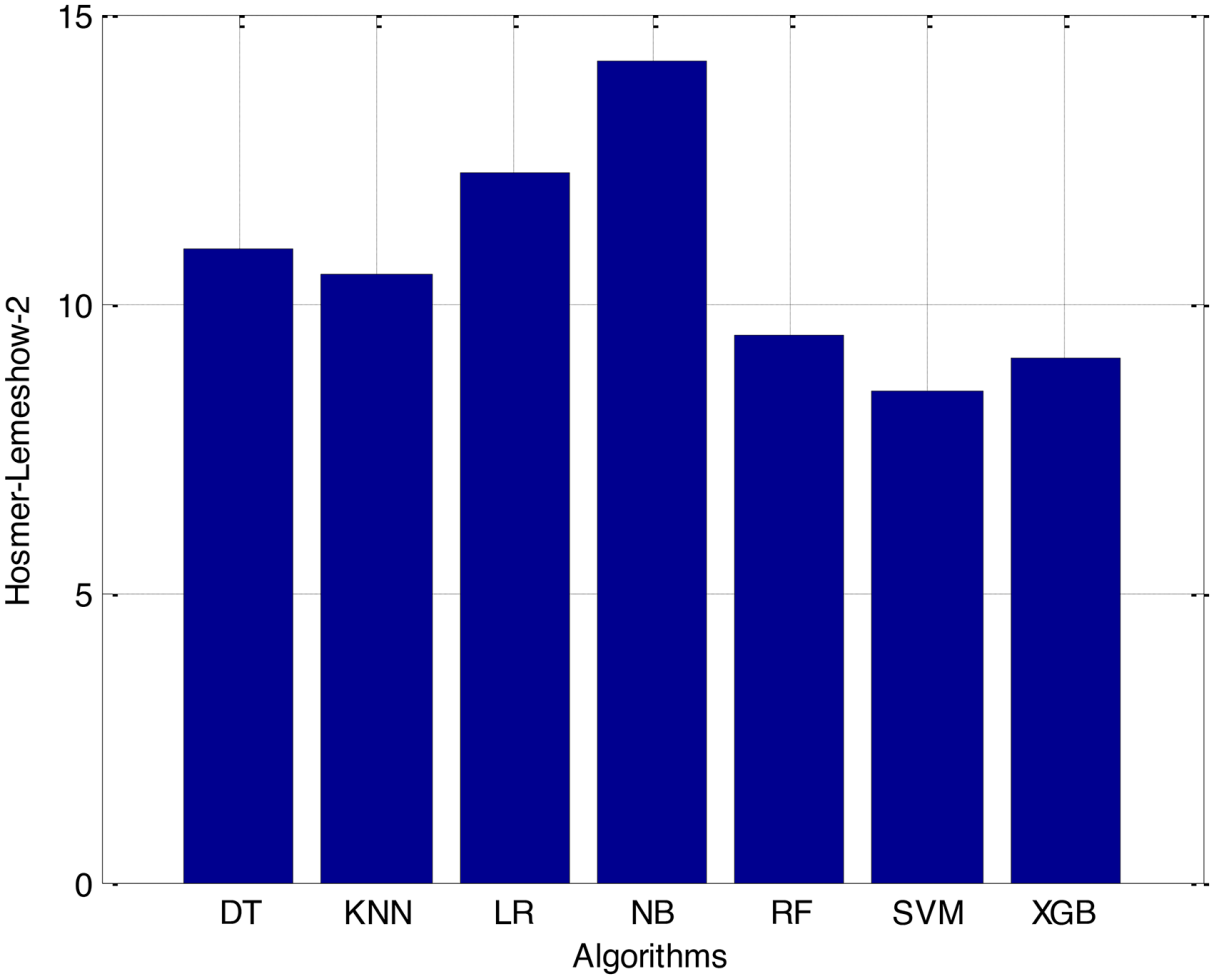
Hosmer-Lemeshow-2.

According to the results of feature selection that was based on RF, the age of the patient was the most significant predictor in the classification of CVD. In this study, it was discovered that certain risk factors, such as smoking and alcohol intake, were not as predictive as previously believed. Previous studies have shown that the synthetic indices BAI and LHR are both very good indicators of cardiovascular disease. Inflammation plays a significant part in the formation of atherosclerotic plaques as well as1 the progression of cardiovascular disease is shown in Figures 6–11.

There is evidence that inflammatory cytokines, such as high-sensitivity CRP and interleukin-6, are associated with an elevated risk of cardiovascular disease. The Hs-CRP inflammatory marker was included in the Reynolds Risk Score in order to account for its role as a potential contributor to cardiovascular disease. hs-CRP has been shown in a number of other epidemiological studies to be an important predictor of CVD. These studies have also shown that hs-CRP acts as a mediator in the pathogenesis of vascular disease and is a marker of endothelial dysfunction. These findings are consistent with the findings of the aforementioned studies. It was discovered that Hs-CRP increases the risk of atherosclerotic plaque rupture in addition to destabilising atherosclerotic plaques *via* NO, IL-6, and prostacyclin.

In addition, hs-CRP has been demonstrated to enhance thrombosis and cardiomyocyte apoptosis in response to hypoxia, which provides more support for its position as a risk factor for cardiovascular disease. It has been demonstrated that IL-6 is a factor in the course of atherosclerosis and that it promotes the creation of atherosclerotic plaques. This factor may have contributed to the increase in the number of cases of CVD. Taking statins, which can reduce a person chance of acquiring CVD, is beneficial for a great number of people and can help them avoid developing the condition. In clinical practise, Hs-CRP and IL-6 can be used as biomarkers for the early diagnosis of patients who have an increased likelihood of developing cardiovascular disease.

According to the findings of our study, a decrease in ADP was associated with an increased risk of developing cardiovascular disease. The adipose hormone ADP, which is secreted by adipocytes, has anti-inflammatory effects. These effects manifest themselves as a reduction in the levels of CRP and lymphocyte recruitment in atherosclerotic lesions, a reduction in the expression of TNF-, and an increase in the production of cytokines that are protective against inflammation. On the other hand, there is evidence from a small number of studies that suggests an increase in ADP may assist in avoiding an ischemic stroke. Increased NEFA concentrations have been associated with an increased risk of cardiovascular disease in previous research, and our study came to the same conclusion. The possible effects of NEFA on cardiovascular disease include the potential to exacerbate or worsen a number of illnesses, including type 2 diabetes, hypertension, the metabolic syndrome, and endothelial deterioration, to name a few. Patients can have a lower chance of developing cardiovascular disease if they are treated to have a lower ADP (CVD).

The risk prediction models that are currently being used in CVD domains were built using traditional statistical methodologies, as many studies have revealed. Nevertheless, these models have been proven to be erroneous in external populations. In the field of cardiology, machine learning algorithms have proven to be superior methods for deriving predictions from big datasets that are notoriously difficult to understand. No prior assumptions are made by machine learning algorithms, which means that any data can be used to develop accurate and resilient models. Because of this, ML is able to model more complex relationships between outcomes and predictors, which are typically more challenging to express using standard statistical methods. Discovering interactions between marginal predictors can help improve risk-management strategies when using ML.

Machine learning has the potential to identify new genotypes and phenotypes for a variety of CVDs, as well as novel risk factors for CVDs. All aspects of medical picture recognition, diagnosis, outcome prediction, and prognosis evaluation can be improved with the application of more sophisticated machine learning techniques such as deep learning and artificial neural networks (ANN). It possible that in the future, cardiologists will make better clinical decisions if they use machine learning models rather than the CVD risk stratifications that are currently used. On the other hand, most ML models may be hard for medical professionals to understand and use, which may limit how often they can be used in clinical settings.

## 5. Conclusion

According to the findings of this research, a stacking fusion model-based classifier performs better than individual models on all assessment criteria. This finding suggests that stacking models can combine the benefits of a variety of model types to achieve superior prediction performance. The recommended stacking approach offers improved prediction performance, increased resilience, and increased utility for individuals who are at high risk of developing cardiovascular disease. Hospitals can utilise this information to identify patients who are at a high risk of developing cardiovascular disease and provide them with early clinical intervention in order to reduce that risk. Research in the field of deep learning will benefit from additional data from a large number of medical institutions, which may be used for the development of artificial neural network structures or for the usage of deep learning frameworks in the future. In future work, the other deep learning techniques algorithms can be incorporated into Internet of Things (IoT) environments which helps to achieve more accuracy in terms of result and it can be useful to the hospitals and saving several human life.

## Data availability statement

The raw data supporting the conclusions of this article will be made available by the authors, without undue reservation.

## Author contributions

MA and KS: conceptualization. SS, NV, and MA: methodology and investigation. TU and LA-k: software. MSo, TU, and NA: validation. KA and RK: formal analysis. KA and MA: data curation. SS and NV: writing—original draft preparation. MA, MSo, LA-k, and RK: writing—review and editing. LA-k, NA, and RK: supervision. All authors contributed to the article and approved the submitted version.

## Conflict of interest

The authors declare that the research was conducted in the absence of any commercial or financial relationships that could be construed as a potential conflict of interest.

## Publisher’s note

All claims expressed in this article are solely those of the authors and do not necessarily represent those of their affiliated organizations, or those of the publisher, the editors and the reviewers. Any product that may be evaluated in this article, or claim that may be made by its manufacturer, is not guaranteed or endorsed by the publisher.
